# In Vitro Re-Hardening of Bleached Enamel Using Mineralizing Pastes: Toward Preventing Bacterial Colonization

**DOI:** 10.3390/ma13040818

**Published:** 2020-02-11

**Authors:** Andrea Scribante, Claudio Poggio, Simone Gallo, Paolo Riva, Antonella Cuocci, Manuel Carbone, Carla Renata Arciola, Marco Colombo

**Affiliations:** 1 Department of Clinical-Surgical, Diagnostic and Paediatric Sciences-Section of Dentistry, University of Pavia, 27100 Pavia, Italy; andrea.scribante@unipv.it (A.S.); paoloriva23@yahoo.it (P.R.); antonella.cuocci01@universitadipavia.it (A.C.); carbonemanuel@hotmail.it (M.C.);; 2 Laboratorio di Patologia delle Infezioni Associate all’Impianto, IRCCS Istituto Ortopedico Rizzoli, via di Barbiano 1/10, 40136 Bologna, Italy; 3 Department of Experimental, Diagnostic and Specialty Medicine, University of Bologna, via San Giacomo 14, 40126 Bologna, Italy

**Keywords:** enamel, tooth bleaching, micro hardness, prophylaxis paste, bacterial colonization

## Abstract

The search for materials able to remineralize human hard tissues is a modern medical challenge. In this study, the protective effect on the enamel microhardness by a paste based on hydroxyapatite and sodium fluoride (Remin Pro) was evaluated after two different enamel bleaching procedures. Forty sound human incisors were randomly assigned to different treatments: bleaching with an in-office agent (Perfect Bleach Office+); bleaching with an at-home agent (Perfect Bleach); bleaching with the in-office agent followed by the prophylaxis paste; bleaching with the at-home agent followed by the prophylaxis paste; no treatment (control). Bleaching was performed at 0, 8, 24 and 32 h, followed by a 3-min re-mineralizing treatment in the subgroups designed to receive it. Specimens underwent a micro-hardness tester and a mean Vickers Hardness number was considered for each specimen. ANOVA exhibited significant differences among groups. Post-hoc Tukey testing showed significant micro-hardness decrease after the application of both the two bleaching agents. The treatment with prophylaxis paste significantly increased the micro-hardness values of bleached enamel.

## 1. Introduction

In recent years, the search for a pleasant smile with whiter teeth has become one of the most common desires among patients [1]. Color is considered as an important factor in aesthetics and a chromatic alteration of the tooth is perceived by the patient more quickly than any other anatomical dental modification [2]. This is the reason for the widespread interest in teeth bleaching [3,4], carried out using carbamide or hydrogen peroxide.

It has been demonstrated that teeth whitening brings a great aesthetic benefit for the patients and, with an accurate diagnosis, planning of appropriate procedure and attention to technique, this treatment is a conservative approach to lightening discolored teeth [5] with an efficacy widely reported in literature [6,7]. However, findings concerning the safety of whitening products on the enamel seem to be controversial [8,9].

In fact, several studies have described the negative impact on the integrity of the hard tissues of the tooth [10,11,12,13]. However, no macroscopic changes have been reported, not even when high concentrations of peroxide are used. Conversely, microscopic side effects on the enamel, such as alteration of calcium and phosphate mineral content [14], increased roughness [15], decrease in hardness [16], increased porosity and superficial irregularities [17] have been demonstrated.

Moreover, other negative factors related to bleaching treatments are generally regarded, like the rebound of stains, soft tissues irritation and, above all, tooth sensitivity, which is the most commonly reported side effect [18].

Several studies indicated that using remineralizing agents following bleaching procedures could repair the microscopic side effects. These agents could remineralize early lesions owing to their chemical similarity with tooth minerals. Fluoride application could be effective because it forms fluorapatite on the enamel surface, recovers the enamel hardness and promotes remineralization [19,20], although some authors reported no improvement in fluoride uptake by bleached enamel [21] or even a major number of morphological changes in case of acidulated fluoride gel application after a bleaching treatment with 35% hydrogen peroxide [17].

This is the reason why many other biomimetic materials have been sought to maintain the integrity of the bleached enamel.

Hardening should be useful in preventing infection, as bacteria take advantage of micro-erosions to colonize enamel [22,23]. Similarly, bone micro-fractures give bacteria the chance to find an entrance and form biofilms [24,25]. Once bacteria have colonized the eroded tissues, they concur to exacerbate demineralization. Therefore, even in orthopedics, bone hardening procedures should ensure the reconstructed bone maintains adequate hardness to resist bacterial colonization [26]. Hydroxyapatite and hydroxyapatite-based composites are widely used in dentistry and orthopedics [27,28,29]. They have also confirmed good performances in infection scenarios [30,31].

The present study aimed to evaluate the remineralization effectiveness of a prophylaxis paste (Remin Pro), based on hydroxyapatite and sodium fluoride, after treating enamel with two different kinds of whitening (with professional and home agents). Enamel hardness, after bleaching procedures and treatment with a prophylaxis paste, was monitored with surface micro-hardness measurements. Although not directly tested in the present report, it can be assumed that enamel hardening contributes to the prevention of bacterial colonization along with the antibacterial role of xylitol contained in the product.

The null hypothesis of this study is that the tested product does not perform any protective action after a bleaching treatment and that there is no difference on the mechanical properties of the enamel respectively bleached with a professional and a home whitening agent.

## 2. Materials and Methods

### 2.1. Specimen Preparation

Specimens were prepared from 40 human incisors, extracted for periodontal reasons and free of caries and defects. Patients gave consent for the study which has been approved by the Unit Internal Review Board. After the extraction, soft tissue debris were removed and teeth were accurately evaluated on their labial enamel surface. They were excluded in the case of white spot lesions, caries, hypoplasia, cracks, and any previous treatment with chemical agents. Finally, disinfection was performed by dipping the teeth in a 5% sodium hypochlorite solution for one hour, which could not alter the enamel surface [32,33]. Each specimen was cut at the enamel-dentin junction using a high-speed diamond rotary bur (Komet Brasseler GmbH & Co., Lemgo, Germany) with a water-air spray. The labial surfaces of the crowns were ground under water irrigation using silicon carbide papers (grades 600 to 1200) (3M ESPE, St. Paul, MN, USA) so that the enamel surfaces appeared smooth and flat [34]. The purpose of this process was to remove the highly mineralized aprismatic enamel which might be present in the outer surface of the tooth. This enamel is extremely variable: it is more frequent in deciduous teeth but can be found in permanent teeth, too. As well, it gradually disappears with mastication but can remain in protected areas. Since this layer is more resistant to acid dissolution [35], it was removed so that individual enamel variations were reduced and samples were standardized [36].

Finally, specimens were placed into Teflon molds (Morefluon Industries, Rabale, Navi Mumbai) of 10 mm × 8 mm × 2 mm, placing the labial surface of the teeth upwards, and they were embedded in flowable composite resin (G. ænial Flo X, Gc Dental Products Corp, 2–285 Toriimatsu-cho, Kasugai, Aichi 486-0844, Japan) and polymerized.

### 2.2. Bleaching Procedure and Remineralization

Before starting the bleaching procedure, each one-half tooth was covered with two layers of adhesive tape to avoid any contact with the whitening agent so that each sample had one-half used for the experimentation and the other one-half which served as control. A fine indelible pen was used to mark the transition between the two areas [34].

The bleaching procedure was performed respectively with two products: one professional whitening agent, such as 35% hydrogen peroxide (Perfect Bleach Office+), and one home whitening agent, such as 16% carbamide peroxide (Perfect Bleach). For the remineralization treatment, a prophylaxis paste (Remin Pro) was employed. The characteristics of the products tested in this study, such as chemical composition and manufacturer, are listed in Table 1.

Specimens were randomly assigned to 4 groups of 10 samples and each group was further composed of two subgroups of 5 specimens each, as listed below:

— subgroup 1a: intact enamel (no treatment was done),
— subgroup 1b: enamel + Perfect Bleach Office+,
— subgroup 2a: intact enamel (no treatment was done),
— subgroup 2b: enamel + Perfect Bleach,
— subgroup 3a: intact enamel (no treatment was done),
— subgroup 3b: enamel + Perfect Bleach Office+ + Remin Pro,
— subgroup 4a: intact enamel (no treatment was done),
— subgroup 4b: enamel + Perfect Bleach + Remin Pro.

The only bleaching procedure without the remineralizing treatment was conducted in subgroups 1b and 2b. The bleaching agents were used according to the manufacturer’s instructions and they were directly applied from the mixing tip in a 1 to 2 mm thick layer onto each half labial surface not previously covered with the adhesive tape [37]. As to the former subgroup, Perfect Bleach Office+ was homogeneously distributed on the teeth surface and moved around with an instrument every 5 min without brushing to stress its demineralizing potential and to reduce the buffering effect from the ions dissolved from the enamel surface [38]. After 15 min, the product was wiped off with distilled water washing. Four intervals of the bleaching procedure were carried out at 0, 8, 24 and 36 h, for a total of an hour of exposure to the professional whitening agent; between two consecutive phases of the experimentation, teeth were stored in artificial saliva (pH 7.0, 14.4 mM NaCl; 16.1 mM KCl; 0.0003 mM Cl_2.6_H_20_; 2.9 mM K_2_HPO_4_; 1.0 mM CaCl_2.2_H_2_O; 0.10 g/100 mL sodium carboxymethylcellulose) [37].

Conversely, in subgroup 2b, Perfect Bleach was homogeneously distributed and left on the labial surface of the teeth for 2 h without brushing the teeth surface. After that, the product was wiped off with distilled water. Even in this subgroup, four intervals of the bleaching procedure were carried out at 0, 8, 24 and 36 h, for a total 8 h of exposure to the home whitening agent, and teeth were stored in artificial saliva between two consecutive bleaching procedures.

Regarding the remineralizing treatment carried out exclusively in subgroups 3b and 4b, the tested agent (1, 5 mL) was applied without brushing after each whitening challenge (performed with Perfect Bleach Office+ in subgroup 3b and Perfect Bleach in subgroup 4b, exactly as previously described for subgroups 1b and 2b, respectively) and the product was then removed with distilled water washing after 3 min of application. Even in these subgroups, teeth were stored in artificial saliva between each remineralizing treatment and the subsequent bleaching challenge. At the end of the experimentation, adhesive tape was removed from all the samples and these could dry in air [34] before being evaluated regarding their surface micro-hardness.

### 2.3. Surface Micro-Hardness (SMH) Measurements

The micro-hardness of the enamel surface was measured after the treatment. The micro-hardness tester (Galileo Isoscan HV1 OD; LTF SpA, Antegnate, BG, Italy) was composed of a Vickers diamond indenter which was applied to each specimen with a load of 100 g for 10 s. Five indentations were realized on each specimen’s surface; they were equally placed in a circle, with a distance of at least 0.5 mm to adjacent indentations [39]. The diagonal indentation length was measured with a 40× objective lens. The Vickers Hardness number (VHN) in kgp/mm^2^ was calculated using the following Equation: HV = 1.854 P/d2, where P is the load in kgf and d is the average length in mm of the diagonals. For a given specimen, a single hardness value was reported, resulting from the mean of the five hardness values calculated.

Statistical analysis was performed with R software (R version 3.1.3, R Development Core Team, R Foundation for Statistical Computing, Wien, Austria). Descriptive statistics including the mean, standard deviation, median, minimum and maximum values were calculated for all groups.

The normality of the data was calculated using the Kolmogorov–Smirnov test. Analysis of variance (ANOVA) was applied to determine whether significant differences in micro hardness values existed among the various groups. Tukey’s test was assessed post-hoc. Significance for all statistical tests was predetermined at *p* < 0.05.

## 3. Results

Descriptive statistics of the micro-hardness values of the enamel for each subgroup are presented in Table 2.

**Figure 1:**
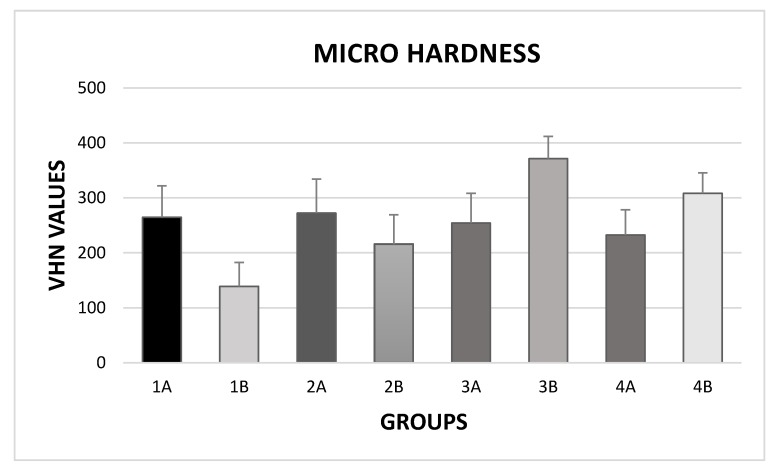
Micro hardness values (Mean and SD) of the different groups.

ANOVA showed the presence of significant differences among the various groups (*p* < 0.0001). As shown in Figure 1, post-hoc Tukey testing showed that both the application of 35% H_2_O_2_ and 16% CH_6_N_2_O_3_ resulted in significantly decreased micro-hardness values (respectively with *p* < 0.001 and *p* < 0.05).

On the other hand, after the treatment with the two bleaching agents, the application of the prophylaxis paste containing hydroxyapatite and sodium fluoride significantly increased micro-hardness values (*p* < 0.05). This was more relevant if enamel had been bleached with 35% H_2_O_2_. Groups with intact enamel showed no significant difference among them (*p* > 0.05).

## 4. Discussion

The aesthetic advantage of teeth whitening is certainly recognized, but several negative effects have been discussed. Although no macroscopic changes of the enamel surface have been reported for this treatment, several studies in the literature focused on the microscopic alterations. It’s important to underline that many of these works have foreseen a single and long-lasting application of the peroxides on the teeth, differently from the manufacturer’s recommendations and to the common use in clinical practice as well [40]. On the contrary, in the present study different bleaching challenges were performed, and the manufacturer’s instructions were strictly followed. All the products were applied onto the teeth surface without brushing in order to exclude the mechanical effect which some authors reported to be a more relevant factor in increasing enamel roughness [41].

Furthermore, among the experimentation phases, specimens were stored in artificial saliva in order to simulate the physiological oral conditions [42], in which saliva contributes to the neutralization of the acids and represents a source of inorganic ions which take part to the remineralization [43].

Many products have been proposed to contrast mineral loss following a bleaching treatment since enamel has no spontaneous capability to repair because of the absence of cells [44,45,46,47]. In a previous study conducted by an author of our group [38], the bleached enamel morphology had been evaluated using scanning electron microscopy after the application of the following agents: a product based on hydroxyapatite and fluoride (Remin Pro), a fluoride varnish (Profluorid Varnish), a casein phosphopeptide-amorphous calcium phosphate (CPP-ACP) paste (Tooth Mousse) and a casein phosphopeptide-amorphous calcium phosphate fluoride (CPP-ACPF) paste (MI Paste Plus). Scanning Electron Microscopy (SEM) microphotographs revealed that the formation of a protective layer on bleached enamel occurred in a different way among these agents. Only CPP-ACP paste and CPP-ACPF paste formed a homogeneous apatic layer on enamel surface, whereas areas of demineralization still appeared if Remin Pro or Profluorid Varnish had been used.

Considering the mechanical properties of the enamel after the application of prophylaxis pastes, the purpose of this study was to determine the effects of Remin Pro on enamel micro-hardness following two different bleaching treatments (with professional and home agents).

The null hypothesis of the study has been rejected. In fact, the remineralization treatment with Remin Pro (subgroups 3b and 4b) significantly increased micro-hardness values compared with the bleached enamel that did not receive it (subgroups 1b and 2b). This result is in accordance with other studies [48].

As expected, the subgroups 1b (enamel + Perfect Bleach Office+) and 2b (enamel + Perfect Bleach) showed that the bleaching agents cause changes on the enamel structure with a reduction of the hardness number, especially using the professional whitening agent at 35% hydrogen peroxide which was obviously more aggressive than the home whitening at 16% carbamide peroxide (which corresponds to 6% hydrogen peroxide). According to the literature, this study undoubtedly confirmed that teeth whitening procedures led to enamel alterations [49,50,51]. In particular, the oxide-reduction of the peroxides seems to be related to demineralization and dissolution of the organic dental matrix (protein) [5,16,52]; although the organic component represents a minor part of the enamel structure (1%), its action is essential for its integrity since it glues the mineral crystals of the enamel together, preventing micro-hardness loss [53]. According to the manufacturer, Perfect Bleach has a pH value of 6 at 20 °C, whereas Perfect Bleach Office+ has a value of 7.3 at the same temperature. With regards to the former, carbamide peroxide releases hydrogen peroxide (1/3) but also urea (2/3) which is rapidly converted into carbon dioxide and ammonia. Urea degrades the enamel organic matrix and causes the formation of free spaces which allow hydrogen peroxide to diffuse throughout the enamel until the dentin-enamel junction [8].

This study also revealed that the micro-hardness values of bleached enamel treated with Remin Pro appeared significantly higher even than the ones registered in the three subgroups of intact enamel (no treatment done), suggesting that the use of a prophylaxis paste is a key point after bleaching procedures increasing enamel hardening. Another result of this study was that the protective effect of the tested product was different considering how bleaching treatment had been performed. In fact, the remineralization process was more powerful in the subgroup 3b, treated with the professional whitening agent, rather than the home agent used in subgroup 4b. This might be explained considering that hydroxyapatite could be more effective in penetrating through the bleached enamel if it has been treated with a higher concentration of peroxide. Similarly, fluoride penetration has been described by some authors to be facilitated in case of increased enamel erosion [54].

## 5. Conclusions

Several agents have been proposed for their protective effect after whitening treatments. Despite the limitations of this in vitro study, a remineralizing treatment with a prophylaxis paste based on hydroxyapatite and sodium fluoride has been shown to be effective in increasing the enamel micro-hardness values after a bleaching treatment, especially if this latter has been conducted using a professional product (35% hydrogen peroxide) rather than a home formulation (16% carbamide peroxide). The application of Remin Pro after a bleaching treatment is always advisable since the enamel micro-hardness increases massively, even reaching higher values than teeth that didn’t undergo a bleaching procedure.

## Figures and Tables

**Table 1:** Characteristics of the materials used in this study.

Group	Material	Type	Composition	Manufactured	Batch Number
1	Perfect Bleach Office+	Professional whitening agent	Hydrogen peroxide 35% (activated gel)	Voco GmbH, Cuxhaven, Germany	1902200
2	Perfect Bleach	Home whitening agent	Carbamide peroxide 16% (activated gel)	Voco GmbH, Cuxhaven, Germany	1903699
3	Remin Pro	Prophylaxis paste	Hydroxyapatite, sodium fluoride (1450 ppm fluoride), xylitol	Voco GmbH, Cuxhaven, Germany	1809775

**Table 2:** Descriptive statistics (Vickers Hardness number (VHN)) of the different groups.

Group	Enamel	Mean (SD) (kgp/mm^2^)	Minimum Value (kgp/mm^2^)	Median (kgp/mm^2^)	Maximum Value (kgp/mm^2^)
1a	Intact	264.74 (57.10) ^a^	151.70	263.18	365.00
1b	H_2_O_2_ 35%	138.96 (43.48) ^b^	64.20	136.80	231.20
2a	Intact	272.21 (61.68) ^a,f^	112.50	292.05	335.90
2b	CH_6_N_2_O_3_ 16%	215.79 (53.13) ^c,e^	92.30	222.35	313.60
3a	Intact	254.26 (53.95) ^a,e^	146.80	252.45	350.50
3b	H_2_O_2_ 35% + Hydroxyapatite	371.08 (40.70) ^d^	293.50	369.70	453.70
4a	Intact	232.29 (46.04) ^a,e^	150.10	229.75	335.20
4b	CH_6_N_2_O_3_ 16% + Hydroxyapatite	308.24 (36.97) ^d,f^	240.30	303.70	382.10

SD: standard deviation. Significant difference at *p* < 0.05. Superscript letters (a, b, c, d, e and f) have been used to indicate statistical results: different letters among the groups indicate significant difference in micro-hardness among the groups.
